# Extracorporeal Membrane Oxygenation (ECMO) Use as Supportive Therapy in a Patient With Acute Respiratory Distress Syndrome Due to Rupture of a Pulmonary Hydatid Cyst

**DOI:** 10.7759/cureus.55223

**Published:** 2024-02-29

**Authors:** Ayman F Yousef, Ahmed A Alzahrani, Malaz S Younis, Muna S Gumaa Albashari, Mohammed S Younis

**Affiliations:** 1 Thoracic Surgery, King Saud Medical City, Riyadh, SAU; 2 Surgery, King Saud Medical City, Riyadh, SAU; 3 College of Medicine, Alfaisal University, Riyadh, SAU; 4 Internal Medicine, University of Medical Sciences and Technology, Khartoum, SDN

**Keywords:** acute respiratory distress syndrome (ards), acute respiratory distress syndrome, ecmo, cyst hydatid, echinococcosis, pulmonary hydatid disease

## Abstract

Pulmonary echinococcosis is a parasitic infection that accounts for 20% of the infected cases with echinococcosis. Patients may present after a cyst rupture associated with a variety of complications, including acute respiratory distress syndrome (ARDS). Extracorporeal membrane oxygenation (ECMO) is known as supportive therapy for patients with respiratory and cardiac failure, including ARDS associated with multiple causes. Parasitic infection associated with ARDS due to cyst rupture managed with ECMO as bridging to definitive surgical intervention is documented in two previous case reports only. Here, we are presenting a 21-year-old female with a pulmonary hydatid cyst complicated by ARDS and managed with ECMO.

## Introduction

Echinococcosis, also known as hydatid disease, is a parasitic infection caused by cestodes belonging to the genus *Echinococcus*. It is characterized by the formation of cysts primarily in the liver and lungs, although other organs may also be affected. Designated by the World Health Organization (WHO) as one of the 17 neglected tropical diseases targeted for eradication by 2050, echinococcosis represents a significant global public health concern [1]. Human hosts are typically considered accidental intermediate hosts in the parasite's life cycle. Pulmonary echinococcosis constitutes approximately 20% of all cases, with the lungs being the second most commonly affected organ after the liver. While isolated cysts may occur, 20% to 30% of cases involve the development of multiple cysts [2]. Treatment primarily involves surgical intervention, often supplemented with medical management utilizing benzimidazoles, with albendazole being the preferred medication [3].

Extracorporeal membrane oxygenation (ECMO) therapy serves as a supportive measure for cases of respiratory and cardiac failure. This therapy employs mechanical extracorporeal circuits to directly oxygenate the blood and remove carbon dioxide, reducing reliance on invasive mechanical ventilation. This approach minimizes the risk of barotrauma and allows a compromised lung to rest [4]. ECMO has demonstrated effectiveness as a bridge to recovery in conditions such as acute respiratory distress syndrome (ARDS) resulting from bacterial or viral infections like COVID-19 [5,6], as well as in providing support for lung transplant recipients [7]. While ECMO has been extensively utilized in various contexts, its application in treating ARDS associated with parasitic infections like echinococcosis remains limited, with only two documented cases in the literature thus far [8,9]. This presents an opportunity to explore the feasibility and efficacy of ECMO in such scenarios. In this report, we present a case of ARDS secondary to the rupture of a pulmonary hydatid cyst managed to utilize ECMO.

## Case presentation

**Figure 1:**
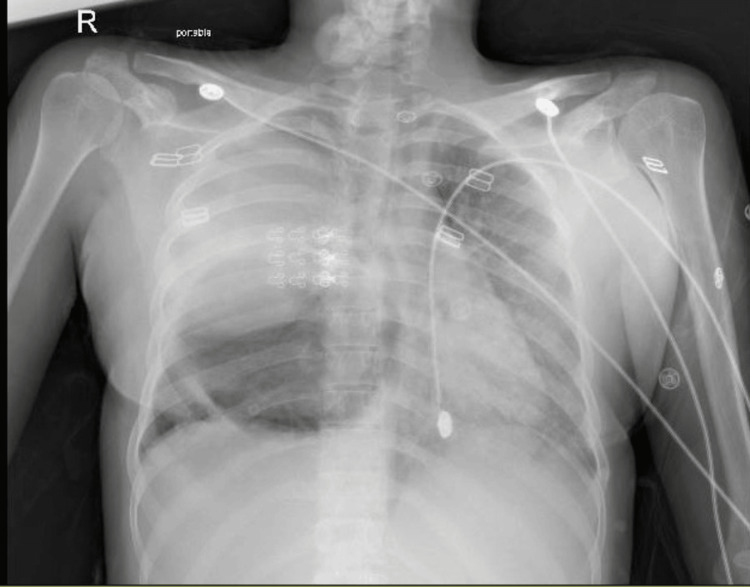
Chest X-ray, anteroposterior (AP) view: large cyst on the field of the right lung.

**Figure 2:**
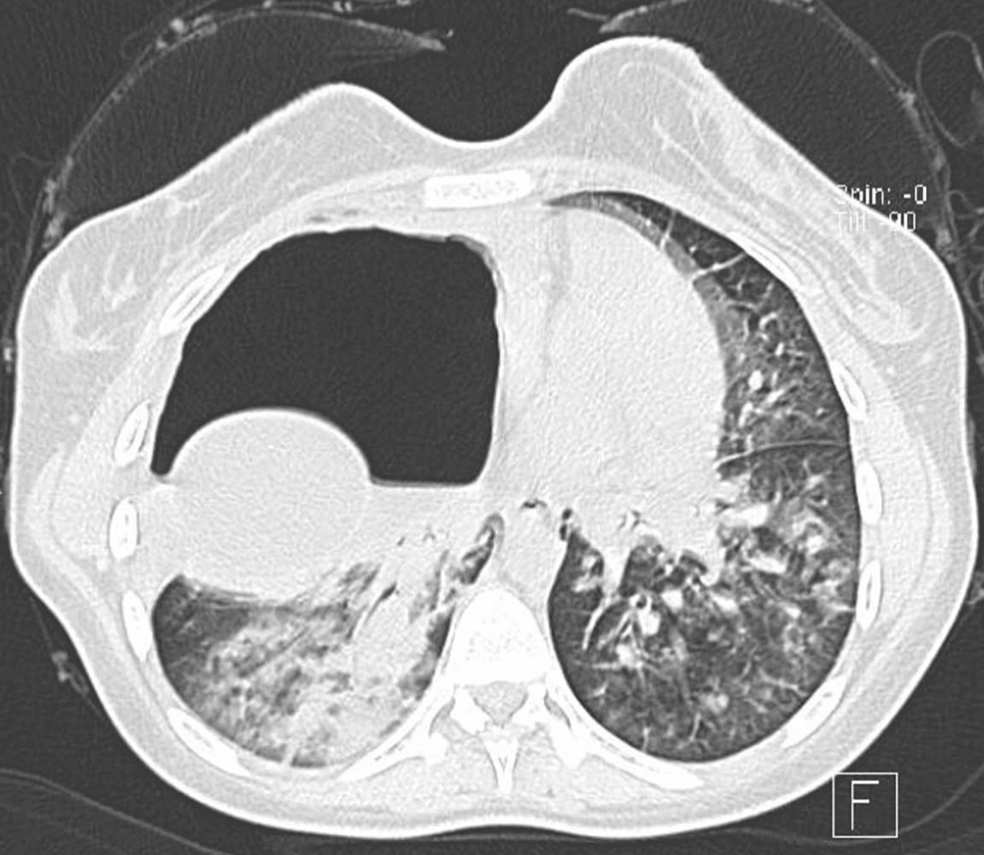
CT chest with contrast, axial plane: bilateral ground glass opacity with a ruptured hydatid cyst in the right lung.

A 21-year-old Syrian female was urgently transferred to our hospital's emergency room from another medical facility. She presented with severe shortness of breath, dry cough, and fever persisting for 10 days. According to her father, she previously worked on a farm in Syria, primarily tending to domestic animals, particularly cattle, and often accompanying dogs while herding. Initial blood investigations that were done at the previous hospital, including complete blood count (CBC), liver function tests, renal function tests, lipase, electrolyte panel, glucose, and C-reactive protein (CRP), were within normal limits except for leukocytosis. A chest X-ray revealed a significant opacity with a cavitary lesion on the right side, causing displacement of the mediastinum. Given the clinical and radiological findings, a computed tomography (CT) scan confirmed the presence of pneumonia and hydatid cysts, prompting her transfer. Despite receiving oxygen at 15 L/min via a non-rebreather face mask before the transfer, her condition deteriorated, necessitating intubation. On arrival, she was tachycardic with an oxygen saturation of 65% on a fraction of inspired oxygen (FiO2) of 100%, positive end-expiratory pressure (PEEP) of 12, and sedated with fentanyl and midazolam. Despite intubation, her oxygen saturation remained below 70%, prompting confirmation of tube position via chest X-ray. Additionally, confirmation of cuff leak was done with no cuff leak noted. Subsequent intubation with another tube to rule out a blocked endotracheal tube did not improve her condition. As her oxygenation continued to deteriorate, the decision was made to initiate ECMO support. Physical examination revealed decreased air entry with scattered rhonchi on the right side of the chest. CBC showed leukocytosis of 40×10^9/L, hemoglobin of 11 g/dl, and platelet count of 497×10^9/L. A chest X-ray upon admission revealed a large, well-defined cyst in the right lung field (Figure 1). Initial CT of the chest and abdomen demonstrated bilateral ground glass opacities and infiltrates, with a ruptured hydatid cyst in the right lung and two large hydatid cysts in the liver (Figure 2).

The patient was then started on intravenous dextrose 5% in ½ normal saline, enoxaparin, omeprazole, albendazole, vancomycin, and imipenem. Upon admission to the ICU, the ARDS protocol was initiated. The endotracheal tube was replaced with a double-lumen tube, with the right side clamped to isolate the diseased lung, and venous-to-venous ECMO was instituted. Blood culture and bronchoalveolar lavage revealed *Klebsiella pneumoniae* (carbapenem-resistant *Enterobacterales* (CRE)) and *Acinetobacter baumannii *(multidrug-resistant organisms (MDROs)). Consequently, vancomycin and imipenem were discontinued, and ceftazidime, avibactam, and colistin were initiated.

**Figure 3:**
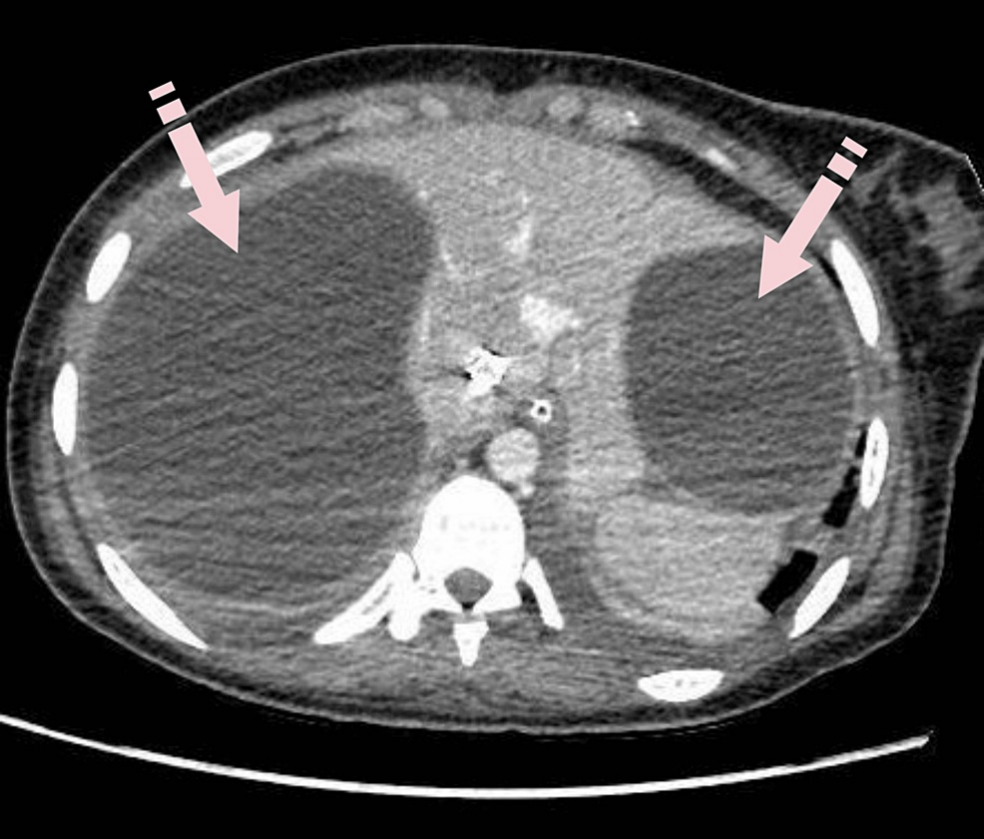
CT of the abdomen with contrast, axial plane: two subcapsular hepatic cysts compressing the right and the left hepatic lobes.

Repeat CT of the chest and abdomen with intravenous contrast revealed patchy ground-glass opacities and lower lobe consolidation in the left lung, accompanied by a small pleural effusion. The right lung exhibited complete collapse, with the middle lobe not visualized and a large cystic lesion measuring 9.5 x 7.5 x 8.8 cm noted, along with multiple folded membranes posteriorly. Additionally, moderate right-sided hydropneumothorax was observed. Abdominal CT demonstrated a 14.6 cm x 11 cm cyst in the right hepatic lobe and an 8.8 cm x 7.5 cm subcapsular cystic lesion in the left hepatic lobe (Figure 3).

Ultrasound-guided pleural vent insertion was postponed to avoid the risk of rupturing the daughter cyst. The leukocytosis decreased to 14.8 × 10^3/L, with mild thrombocytopenia of 113 × 10^9/L and a hemoglobin level of 8.3 g/dl. Hepatic function tests revealed alanine aminotransferase (ALT) of 37.6 U/L, aspartate aminotransferase (AST) of 74.6 U/L, and alkaline phosphatase (ALP) of 71 U/L. Preoperatively, two units of packed red blood cells (PRBC) were transfused. Bronchoscopy was performed with proper suctioning of both lungs.

**Figure 4:**
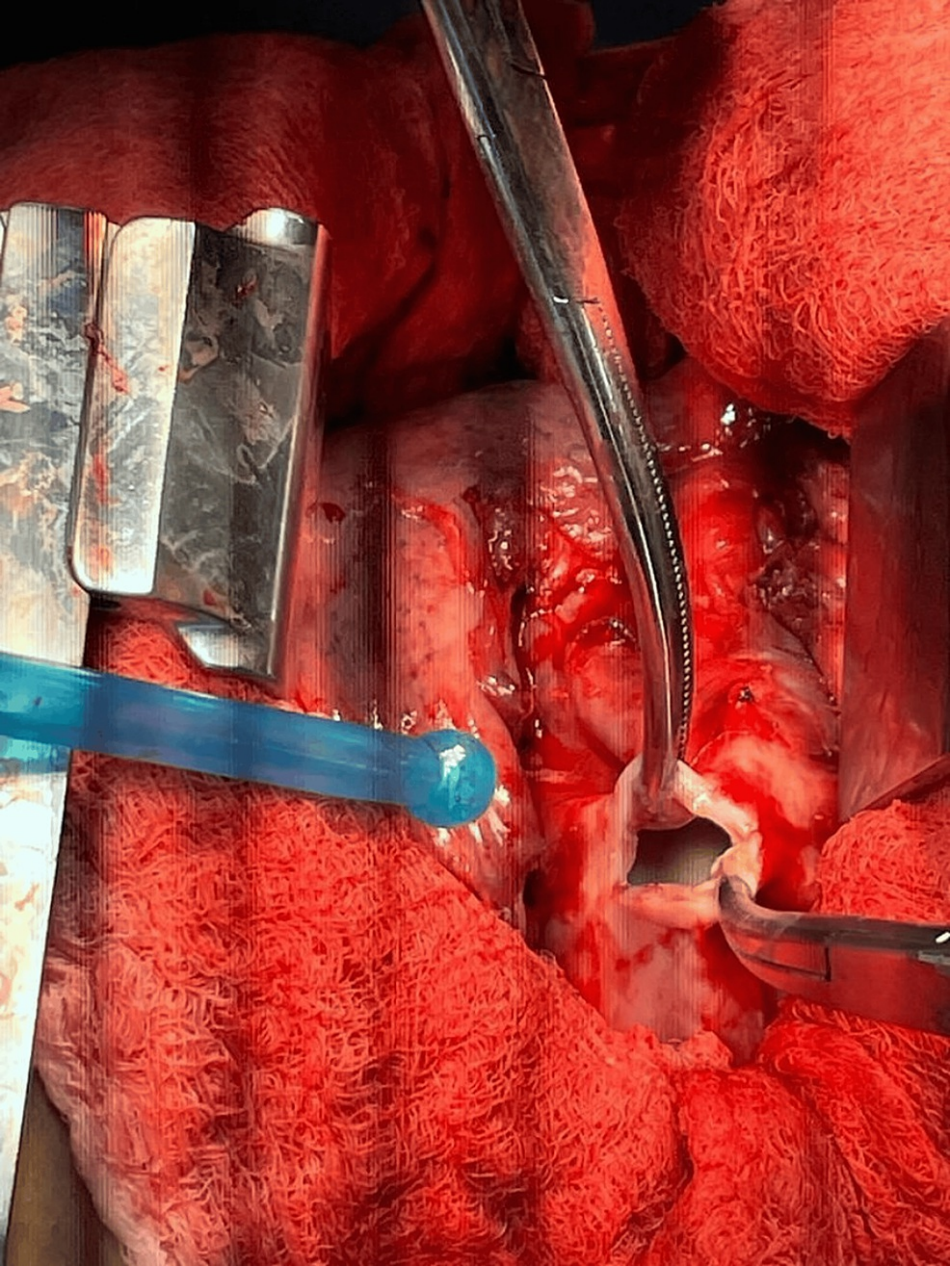
Intraoperative: a small incision was made to drain the fluid from one of the cysts.

The patient underwent a standard right posterolateral thoracotomy (Figure 4), revealing two hydatid cysts in the middle lobe compressing both the upper and lower lobes, one of which was already ruptured. The cysts were excised completely, with a thorough washing of the thoracic cavity using hypertonic saline. Due to significant damage to the middle lobe, a lobectomy was performed. Two intercostal tubes (ICTs) were inserted anteriorly and posteriorly. The patient tolerated the surgery well without intraoperative complications and was transferred to the ICU while still on ECMO in stable condition. Histopathological examination confirmed the presence of hydatid cysts with severe acute inflammation and fibrosis in the resected middle lobe.

Postoperatively, the patient remained hemodynamically stable, sedated, and intubated on ECMO without any episodes of fever. The ICT drained 1 L of haemoserous fluid on the first day, with a subsequent decrease over the following eight days. Chest X-ray revealed satisfactory lung expansion. On the 8th postoperative day, bedside bronchoscopy identified thick secretions, necessitating suctioning. The stump of the lobectomy remained intact, and the anterior ICT was removed. However, the following day, the chest X-ray indicated right lung collapse with increased infiltrates in the right upper lobe. The patient exhibited bloody secretions and blood oozing from the carina and bronchus, prompting endotracheal tube replacement. Despite efforts, oxygen saturation remained low at 83-85% on ECMO (FiO2 of 100%, PEEP of 5). Subsequent bronchoscopy revealed numerous clots, leading to the decision to perform a tracheostomy. Unfortunately, due to a dismal prognosis, the patient was placed on a do-not-resuscitate (DNR) status and succumbed shortly thereafter to the development of disseminated intravascular coagulation (DIC).

## Discussion

Pulmonary echinococcosis presents with diverse clinical manifestations. Patients may exhibit no symptoms at all or they may experience cough, chest pain, hemoptysis, or complications such as ARDS, tension pneumothorax, empyema, hemorrhage, and glomerulonephritis due to cyst rupture [2]. In this particular case, the patient was urgently referred to our hospital due to life-threatening ARDS resulting from a ruptured pulmonary hydatid cyst. CT imaging revealed a complete collapse of the right lung, with a large cyst and multiple folded membranes necessitating surgical intervention. Given the size of the cyst observed on chest X-ray and CT scans upon presentation, the patient likely had an asymptomatic incubation period, which typically lasts between five to 15 years [10]. Initially, the patient was intubated, but despite mechanical ventilation, her condition continued to deteriorate. Subsequently, venovenous ECMO therapy was initiated. While only two previously reported cases in the literature have managed ARDS due to parasitic infection with ECMO, both cases involved ARDS resulting from acute rupture of pulmonary hydatid cysts. In both instances, ECMO proved successful as a supportive therapy, facilitating the transition to definitive surgical intervention and subsequent recovery of the patients [8,9].

Despite the unfortunate outcome for this patient, ECMO therapy played a crucial role in managing ARDS both before and after surgery. It served to stabilize the patient, maintain adequate blood oxygenation, and support organ perfusion preoperatively, enabling postoperative lung recovery and minimizing the risk of barotrauma associated with mechanical ventilation. A meta-analysis conducted in 2019 demonstrated a significant reduction in the 30-day mortality rate for severe ARDS patients treated with ECMO compared to conventional mechanical ventilation, with rates of 31% and 45%, respectively [11]. However, the sudden onset of symptoms, rapid deterioration, documented infection before surgery, and subsequent development of DIC likely contributed to the patient's demise despite aggressive medical and surgical interventions.

Furthermore, the inherent risks associated with the disease and surgical procedures cannot be overlooked, as evidenced by the postoperative mortality rate of 2.2% for hydatid cyst surgery [10]. ECMO itself carries substantial mortality rates, with 30.1% within seven days, 59.8% within a month, and 76.5% within a year. Common ECMO-related complications include acute renal failure, hemorrhage, and ischemic stroke [12]. Notably, 19% of ECMO patients experienced severe hemorrhage, and a small fraction developed fatal pulmonary hemorrhage. These findings underscore the complex risk-benefit profile of ECMO therapy, highlighting the importance of careful patient selection and monitoring to optimize outcomes [11].

In addition, our investigation revealed a notable dearth of studies on the prevalence and impact of hydatid disease in Saudi Arabia. Much of the existing research has focused on livestock in the Qassim region, indicating infection rates of 29.5% in sheep, 14.9% in goats, 6% in camels, and 3.1% in cattle, with an overall livestock infection rate of 11.3%. However, infection rates appear to be lower in native fauna. While there is evidence suggesting an increase in hydatid cystic disease incidences in the country, this trend may be attributed to improved detection methods and expanded medical care coverage in rural areas [13]. Nevertheless, the disease remains a significant public health concern, largely due to limited awareness among clinicians.

The WHO Informal Working Group on Echinococcosis (WHO-IWGE) has issued guidelines for disease management; however, there exists considerable disparity in disease management practices across different regions [14]. This divergence may stem from various factors, including disparities in access to diagnostic tools, such as ultrasound and CT scans, availability of medical treatments, and the absence of surgical intervention facilities in certain areas [15]. Moreover, there is a notable scarcity of evidence-based studies guiding disease management practices. Consequently, patients often present in advanced disease stages with serious complications, underscoring the urgent need for improved disease awareness, standardized management protocols, and enhanced healthcare infrastructure to address this pressing public health issue.

## Conclusions

This case highlights the challenges of managing pulmonary echinococcosis in regions with limited resources and awareness, emphasizing the importance of early recognition and intervention to prevent serious complications. Despite advanced therapies like ECMO, the patient's unfortunate outcome underscores the complexity of the disease. Further research and standardized guidelines are needed to improve patient outcomes, while efforts to enhance disease awareness among clinicians and improve access to diagnostic tools and treatments are crucial in addressing the burden of pulmonary echinococcosis.
